# The impact of environmental information disclosure on enterprises’ green preference of outbound investment: Evidence from China

**DOI:** 10.3389/fpsyg.2022.985727

**Published:** 2022-10-10

**Authors:** Cheng Peng, Wenting Fu, Hui Jiang, Yu Zou

**Affiliations:** ^1^Center for International Business and Economy, School of International Finance and Trade, Sichuan International Studies University, Chongqing, China; ^2^School of Economics and Business Administration, Chongqing University, Chongqing, China

**Keywords:** environmental information disclosure, green preference of outbound investment, green technology innovation, media attention, mediating mechanism

## Abstract

China is accelerating green and low-carbon transformation and promoting high-quality economic development. To effectively contribute to the improvement of global environmental quality and enhance enterprises’ awareness of environmental responsibility in the process of outbound investment, China strongly advocates that enterprises should comply with the environmental protection laws and regulations of host countries and try to strengthen local environmental protection as investing abroad. However, inadequate attention has been paid to the factors influencing green preferences in corporate outbound investment. Therefore, using a sample of Chinese A-share listed companies from 2008 to 2019, this paper aims to empirically analyze whether the quality of environmental information disclosure (QEID) affects enterprises’ green preference of outbound investment (OIGP), and the influence mechanism of QEID on the enterprises’ OIGP by analyzing the mediating effects of green technology innovation and media attention. It is found that QEID significantly promotes corporate OIGP. In terms of influence mechanism, QEID promotes firms’ OIGP by restraining media attention, while the mediating role of green technology innovation in the effect of QEID on firms’ OIGP is manifested as the masking effect. In addition, it is found that these influence mechanisms are different among enterprises with different property rights and different life cycle stages.

## Introduction

The rapid economic development has made environmental pollution a global issue and countries are becoming more and more aware of its importance of sustainable economic development. Environmental degradation has become a major factor that hinders global sustainable development and endangers human life. Based on the concept of ecological security and green sustainable development, many countries have written carbon peak, carbon neutral and other green development goals into their strategic development plans. After promoting the Kyoto Protocol and the Eco-Innovation Plan, the United Nations and the European Union have further mentioned the 17 Goals for People, Planet and the Post-2015 EU and Global Development Framework to integrate ecological protection and sustainable development into their long-term development strategies. Against such a background, more and more enterprises actively or passively incorporate the concept of green development into their corporate social responsibility system, thus affecting their decision-making behavior, including outbound investment decisions. It is, therefore, necessary to understand and evaluate the foreign investment behavior of enterprises from the perspective of green development.

According to the “Pollution Haven Hypothesis (PHH)” the higher the environmental responsibility a firm assumes in its foreign investments, the higher the costs it incurs. Consequently, companies will not initiatively take responsibility for environmental protection, resulting in their choice in the moderate degree of greenness of their foreign investment to avoid excessive cost burden. Thus, enterprises are usually inclined to invest in countries with low environmental requirements. However, companies will appropriately increase their investment in countries or regions with high green requirements due to the fact that investors, the public and the government will lower their evaluation of a company according to its investment behavior, and even reduce their support in various ways (Peng and Jiang, 2021). A study of U.S. firms investing in China has demonstrated that those with greater environmental competence choose to invest in Chinese provinces with stricter environmental regulations (Bu and Wagner, 2016). In this way, companies can demonstrate their strong environmental protection capabilities and environmental responsibilities, thus showing a good image and obtaining more external support. In this sense, environmental information disclosure, as an important source of information for the external market to understand the efficiency and effectiveness of corporate sustainable development strategy (Deegan, 2002; D'Amico et al., 2016), should be able to affect stakeholders’ decision, including investors, creditors and government agencies, on how to support the enterprise by revealing the corporate utilization of environmental resources and the management of environmental pollution, which would further influence the investment behavior of the enterprise.

To effectively reduce the environmental pollution of enterprises and strengthen their responsibility for environmental protection, China has promulgated the Measures on Environmental Information Disclosure since 2007, which stipulates the ways and items of environmental information that firms should disclose, marking the way to legalization of environmental information disclosure. With the promulgation and implementation of the new Environmental Protection Law in 2015 and the Evaluation Report on Environmental Responsibility Information Disclosure of Chinese Listed Companies released in 2017, the overall level of green information disclosure of listed companies in China has been improved to a greater extent. However, existing research on the quality of environmental information disclosure (QEID) focuses on its influencing factors, arguing that both internal factors including corporate governance and internal control (Yan and Chen, 2017), and external factors such as media opinion, government policies, and the intensity of environmental regulations (Fang and Guo, 2018) have a significant impact on the quality of corporate environmental information disclosure. Other scholars are more concerned about the economic consequences of QEID, arguing that QEID can significantly affect firm value (Tang et al., 2021), investment efficiency (Wang et al., 2020), FDI inflows (Shi et al., 2019), firm export size (Fang et al., 2019), export decisions (Lu et al., 2020), domestic value added (Yang and Xie, 2020), and exporters’ markup rate (Li and Fang, 2021). These studies suggest that QEID affects not only firms’ domestic business behavior but also their internationalization behavior. However, how QEID affects firms’ outbound investment behavior including green preferences of outbound investment (OIGP) is to be further explored.

This paper empirically analyzes the impact of QEID on firms’ OIGP by using a sample of Chinese A-share listed companies from 2008 to 2019, and examines the mediating roles played by green technology innovation (GTI) and media attention (MA). In addition, this paper also explores the heterogeneous performance of firms with different property rights and at different life cycle stages. The possible contributions of this paper are: (1) unlike previous studies that focus on the economic consequences of QEID, this paper studies the impact of QEID on firms’ OIGP, which expands the relevant research on QEID and provides empirical evidence for the further improvement of environmental information disclosure system. (2) This study investigates the impact of QEID on firms’ OIGP from two perspectives: green technology innovation and media attention, to provide a reference for optimizing enterprises’ outward investment decisions, especially in the context of “carbon peaking and carbon neutrality goals.”

This paper is structured as follows. In the next section, we present the theoretical analysis as well as the research hypotheses. We then describe the research design, including sample selection, data sources, variable measures, and model design, to empirically demonstrate the impact of the quality of environmental information disclosure on enterprises’ green preference of multinational investment. Finally, we discuss the empirical results and draw conclusions, and explore the potential implications of this study.

## Theory and research hypothesis

### Environmental information disclosure and enterprises’ OIGP

Enterprise environmental information disclosure refers to the relevant behavior of an enterprise to disclose the environmental information related to the company to investors and the public in a certain way in accordance with the laws, regulations, administrative rules of the competent securities authority and relevant regulations of the stock exchange and other regulatory bodies during the process of securities issuance, listing and trading. QEID not only reflects enterprises’ compliance and implementation of the environmental information disclosure system, but also improves the public’s understanding of the behavior of listed companies and helps investors, creditors, and government agencies evaluate the risks, economic benefits, and sustainability of enterprises based on the degree of consistency between their business activities and environmental regulations. Therefore, QEID can send a positive signal to the market through the signal mechanism, thereby alleviating the financing constraints of enterprises, reducing their financing costs, increasing their capital supply, and providing strong support for foreign investment in green projects. Although the PHH argues that businesses face higher costs and financial pressures to invest in countries with strong environmental regulations, resulting in rational firms being more inclined to invest in countries or regions with lax environmental regulations (Bommer, 1999; Pavelin and Porter, 2011), businesses with high QEID generally have access to bank loans with lower interest rates and longer maturities (Allen and Gordon, 2010) and thus higher corporate value (Yang et al., 2020), which alleviates the concerns of enterprises to a certain extent and increases their willingness to invest in countries with sound environmental regulations, indicating that enterprises may have a stronger green preference in their outbound investment. Moreover, high QEID can enhance the exposure of corporate behavior and increase pressures from external supervision, thus urging listed companies to adjust some of their behaviors accordingly. Investment methods or projects that meet the expectations of the public opinion and external supervision can increase the investment efficiency of enterprises (Wang et al., 2020), improve the market position and firm value (Broadstock et al., 2018; Haque and Ntim, 2020), and ultimately achieve a better integration of their economic and environmental benefits. Apparently, in the scenario of the overall increasing global environmental awareness, strengthening OIGP is more in line with public expectations. Therefore, with stricter environmental information disclosure requirements, businesses usually try their best to increase their OIGP in an attempt to send more positive signals to the society, create a good business environment for themselves and enhance their competitiveness.

Based on the above analysis, it proposes the following hypothesis:

H1: the improvement of QEID of firms will promote their OIGP.

### Environmental information disclosure, GTI and enterprises’ OIGP

Green technology innovation (GTI) refers to a kind of technological innovation in line with sustainable development, covering environment-friendly technology, energy-saving technology and innovation of renewable energy technology, etc. It is the primary driving force leading the green development of enterprises. Enterprises are motivated to make more positive environmental management decisions through GTI in the face of institutional pressure from the government and the public to obtain a higher market evaluation (Wang and Ning, 2020). Therefore, environmental information disclosure, as an effective way for the government and the public to monitor firms, can effectively promote their GTI by exerting some pressure on them. Moreover, the academia generally agrees that the innovative behavior of enterprises highly depends on social trust, and establishing good social trust is conducive to the raising of enterprises innovation funds (Li et al., 2020). As subjects of corporate social responsibility, firms can convey positive environmental information about their production and operation to the outside world and establish an image with a high sense of social responsibility with positive environmental information disclosure, which can help them obtain more financial support for their innovation activities (Zhan and Hou, 2021). Furthermore, environmental information disclosure mitigates the information asymmetry between investors and the enterprises, and enables investors to better measure the potential risks and future opportunities of enterprises and reduce their investment risks (Healy and Palepu, 2001; Dhaliwal et al., 2011), thus helping enterprises to raise funds and providing important support for their innovation.

Enterprises can gradually establish differentiated competitive advantages by actively pursuing green innovation while enhancing the favor of consumers with strong environmental awareness and ultimately achieving their goal to expand their market share by stimulating new market demand (Xie et al., 2019). The higher the degree of green in a country, the stronger the environmental protection consciousness of its consumers. Consumers hope to get products that meet their own needs without causing excessive consumption of resources and environmental damage and are willing to bear more environmental premiums to achieve certain ecological compensation, thereby facilitating their environmental protection objectives.This market is undoubtedly more attractive to green innovative enterprises. Moreover, if foreign companies have more efficient technology and less pollution per unit output, their pollution control cost will be lower than that of others under more strict environmental regulations, so that they can have greater cost advantages over their domestic competitors in the host country (Dijkstra et al., 2011). These companies may prefer to invest in countries with a high degree of green compared to those with a low degree of green. In addition, these countries with strict environmental regulations and strong environmental awareness tend to put in place policies to attract these environmentally friendly companies that are active in green innovation to invest in their countries, so as to maintain and improve their green performance.

Based on the above analysis, hypothesis 2 is proposed as follows:

H2: QEID promotes enterprises’ OIGP by facilitating GTI.

### Environmental information disclosure, MA and enterprises’ OIGP

The theory of information asymmetry assumes there is a serious information asymmetry between enterprises and the public in market economic activities, which affects investors’ decision-making. An open media allows for the free flow of information in a market economy, which helps to undermine the information advantage of informed traders, reduce information asymmetry and better fulfill the monitoring function of media attention (Du et al., 2010). In this case, environmental information disclosure functions as an important indicator for investors to measure the attitude to which enterprises take social responsibility and the level of environmental governance (Tao et al., 2020). The media, as an important bridge between the public and enterprises, can influence the green innovation behavior of enterprises by obtaining corporate environmental information and guiding public perception and evaluation (Chen et al., 2018). However, the media is characterized by its pursuit of sensational effects. Influenced by reputation factors, journalists tend to choose sensational news for reporting because reporting sensational news can earn them an international reputation and wide attention, and provide them with greater opportunities for their career development (Wen et al., 2014). Therefore, the media is more interested in the negative news of enterprises and inclined to report their environmental infringement news. Once a major pollution accident occurs in an enterprise, it will become the fodder for the media to compete for coverage. It can be, therefore, assumed that the higher the quality of green information disclosure, the less likely it is to attract public attention because it does not have high reporting value, and the media attention will then decline.

Changes in corporate decision making in response to high intensity media attention are not necessarily positive. On the one hand, the company may actively respond to issues of public concern, and strive to maintain its social reputation, so that its behavior conforms to social values, thereby improving its own governance. On the other hand, the enterprise may take more short-sighted measures to mitigate the current negative impact due to the public opinion pressure caused by the high-intensity media attention. Under such circumstances, to address the intense public pressure and reduce the negative impact of media attention, enterprises are likely to adopt some “greenwashing behavior” to divert public attention, such as false propaganda, emission fraud (Pan et al., 2019), or earning management (Yang et al., 2017). However, in any case above, the enterprise needs to increase funds allocation to eliminate the negative impact of media attention, which, to some extent, crowd out investment in host countries with high environmental requirements and high environmental costs. In addition, environmentally conscious consumers in host countries with high environmental requirements are more sensitive to negative reports and more likely to lose confidence in these enterprises, which ultimately limits their future development in the region. Since high-intensity media attention may weaken enterprises’ OIGP, environmental information disclosure is likely to strengthen the enterprises’ OIGP by suppressing media attention.

Based on the above analysis, hypothesis 3 is put forward as follows:

H3: QEID promotes enterprises' OIGP by suppressing MA.

### Environmental information disclosure, enterprises’ OIGP and different property rights

In China, enterprises with different property rights differ in terms of their business objectives, social functions, internal controls and the policy supports they receive from the government. State-owned enterprises (SOEs) are in a leading position in the economy, and the state has given them the important mission of maintaining social and economic stability and sustainable development. For this reason, state-owned enterprises need to undertake more missions including social development, and their business models and objectives should be more in line with the expectations of the government and the public. Therefore, compared with non-state-owned enterprises, state-owned enterprises obviously face greater pressure on environmental information disclosure and environmental protection. In addition, from the perspective of legitimacy, the level of corporate legitimacy reflects the extent to which the company fulfills the social contract. As the vanguard of the reform of the national economic system, the state-owned enterprises usually assume the role of being the first to try when China implements the reform of the environmental information disclosure system. Furthermore, the state-owned enterprises always attract more attention from the pubic and the level of corporate legitimacy is easy to arouse public interest. SOEs receive more attention from stakeholders and are subject to greater pressure of environmental protection (Bansal and Clelland, 2004; Cormier et al., 2011) since they generally have higher corporate legitimacy and higher quality requirements for environmental information disclosure than non-SOEs. So, it is generally believed that SOEs fulfill more social responsibilities, and thus higher quality of environmental information disclosure and stronger green preference for outbound investment.

Based on the above analysis, hypothesis 4 is put forward as follows:

H4: For firms with different ownership properties, the impact of QEID on firms' OIGP and its influence mechanism is different.

### Environmental information disclosure, enterprises’ OIGP and different life cycle stages

The enterprise life cycle theory divides the existence of enterprises into five stages: birth, growth, maturity, decline, and death, and the development characteristics, production and management strategies, and governance models of enterprises in different life cycle stages are different (Yang and Huang, 2021). For growth stage enterprises, their own capital, technology, and experience are relatively scarce, and their organizational management model is not yet mature (Chen et al., 2019). Since these enterprises have not yet formed a certain scale, their main business goal is to continuously improve market competitiveness and seize the market initiative to expand their market shares in the shortest time. For enterprises in this stage, the short-term profit target is dominant over other social benefit targets. Enterprises in the growth period pay less attention to environmental protection, thus showing a weak green preference for outbound investment. On the contrary, enterprises in the maturity stage often have strong financial strength and a certain influence on various aspects of society, so they pay more attention to their long-term interests and the maximization of social benefits. As a result, enterprises in maturity stage are more committed to environmental protection as a way to improve their social image and social status. Therefore, enterprises at this stage lay more emphasis on environmental information disclosure and environmental protection, thus demonstrating a strong OIGP. Enterprises in the decline stage may not have time to consider environmental information disclosure because of their deteriorating profitability and their frequently-occured operation problems. For firms in this stage of life cycle, survival is their key issue and they are reluctant to spend too much energy and money to protect environment. Even if they have no choice, they may only fulfill some basic requirements or even fulfil environmental target by fraud. As a result, they will show less OIGP.

Based on the above analysis, it proposes the following hypothesis:

H5: For firms in different phases of life cycle, the impact of QEID on firms’ OIGP and its influence mechanism would be different.

## Research methods

### Data and sample

In this paper, we selected enterprises with multinational investment listed in Shanghai and Shenzhen stock markets from 2008 to 2019 as the research samples and finally obtained 5,602 valid observation samples after excluding ST and *ST enterprises and those taking tax-free islands and tax havens as multinational investment destinations. The relevant data of multinational investment events of enterprises are collected and collated through the annual reports of multinational corporations, and other data of enterprises come from the economic and financial database of CSMAR. The green degree of each country is measured by the data of Environmental Performance Index (EPI) jointed developed by Yale University and Columbia University, and other data of the host countries are selected from the official website of the World Bank. What’s more, the data of GTI and MA comes from the CNRDS database of China.

### Definition of variables

#### Dependent variables

Enterprises’ OIGP is the dependent variable of this paper. Yale University and Columbia University jointly released the environmental performance index EPI. The EPI index scores according to the gap between the performance of each country in each index and the established target. The higher the score, the better the green performance. Therefore, it provides a quantitative analysis basis for economists to analyze the green degree of all countries. In order to improve the stability and accuracy of this indicator, referring to Peng and Jiang (2021), this paper adopts green performance difference (*GPD*) to measure the green degree of each country. It is calculated as follows:


(1) $$GPD=\left(EPI_{\mathrm{other}}-EPI_{\mathrm{China}}\right)/EPI_{\mathrm{China}}$$

Wherein, *EPI_China_* is the environmental performance index of China, and *EPI_other_* is that of other countries or regions. If *GPD* is positive, the degree of greenness of the host country is higher than that of China; if *GPD* is negative, the degree of greenness of the host country is lower than that of China; and the smaller the value is, the lower the green degree of the country is. Through Eq. 1, all the national green performance of the host country involved in the multinational investment of enterprises are integrated from the horizontal value level to measure the degree of enterprises’ OIGP.


(2) $$OIGP_{i,t}=w_{i,j,t}GPD_{i,t}$$

Wherein, *OIGP_i,t_* is the green preference of outbound investment of company *i* in year *t* and is the weighted average of the national green performance of all host countries involved in outbound investment of company *i.* This paper refers to Mihov and Naranjo (2019): divide the number of subsidiaries of company *i* in host country *j* by the total number of overseas subsidiaries owned by company *i* in year *t* as weight *w_i,j,t_*. The larger the *OIGP* value is, the stronger the green preference of the enterprise’s foreign investment behavior is; the smaller the *OIGP* value is, the more inclined the enterprise is to invest in the countries and regions with low green degree.

#### Independent variables

##### Quality of environmental information disclosure

In this paper, based on the practice of Kong et al. (2021), the data on environmental information disclosure in the CSMAR database are classified according to whether it is monetized or not. For monetized information, the index of quantitative disclosure is assigned as 2, the index of qualitative disclosure is assigned as 1, and the index of non-disclosure is assigned as 0. For non-monetized information, the disclosed indicator is assigned a value of 2, and the non-disclosed indicator is assigned a value of 0. Specifically, the indicators in environmental liability disclosure, environmental performance and governance disclosure belong to monetized information, while the indicators in environmental management disclosure, environmental certification disclosure and environmental information disclosure carriers belong to non-monetized information. These two types of information have five aspects and 25 scoring items. *QEID* is obtained by adding the scores of these items to one and logarithmic processing, which comprehensively reflects the *QEID*. The specific scoring items of environmental information disclosure are shown in Table 1.

**Table 1:** Environmental information disclosure index rating form.

Disclosure type	Disclosure items	Score description
Environmental management disclosure	Environmental protection concept	Disclosed:2 points Undisclosed:0 points
	Environmental protection objectives	
	Environmental protection management system	
	Environmental protection education and training	
	Special action for environmental protection	
	Environmental time emergency mechanism	
	Environmental protection honors or awards	
	The “three simultaneous” system	
Environmental certification disclosure	Whether it has passed ISO14001 certification	Disclosed:2 points Undisclosed:0 points
	Whether it has passed ISO09001 certification	
Environmental information disclosure carrier	Annual report of listed companies	Disclosed:2 points Undisclosed:0 points
	Social Responsibility Report	
	Environmental Report	
Disclosure of environmental liabilities	Waste water emission	Quantitative:2 points Qualitative:1 points No description:0 points
	COD emission	
	SO_2_ emission	
	CO_2_ emission	
	Soot and power emissions	
	Industrial solid waste emissions	
Environmental performance and Governance Disclosure	Waste gas emission reduction and treatment	
	Waste water emission reduction and treatment	
	Dust and smoke treatment	
	Utilization and disposal of solid waste	
	Noise,light pollution and radiation control	
	Implementation of cleaner production	

#### Mediating variables

##### Green technology innovation

This paper draws on the measurement method of green technology innovation by Qu (2021). Firstly, the patent information is screened according to the IPC code of seven categories of green patents defined in the International Green Patent Classification List launched by the World Intellectual Property Organization in 2021. After classified, retrieved and summarized in the SIPO patent database according to various conditions, the number of authorized green patents of each company is finally obtained. This kind of green patent after examination can more truly and reliably reflect the green technology innovation ability of enterprises. And while the number of environmentally friendly patents has expanded in recent years, the quality of innovation has declined. This may be because green patent includes green invention, green utility model and green design, while only invention needs to pass substantive review, so only green invention is considered when measuring green technological innovation. Therefore, this paper takes the number of green invention authorization of each company as a measure index of GTI.

##### Media attention

In this paper, MA is defined as the media’s coverage of an enterprise, which is measured by dividing the total number of network news and financial news in newspapers and magazines by 1,000 and adding 1 to the logarithm.

#### Control variables

In this paper, the control variables draw mainly from Peng and Jiang (2021) to control for the effect of QEID on firms’ OIGP at both the host country and firm levels. These variables include: *LnGdpp*, *LnLAB*, *Roa*, *Foreign*, *Lev*, *Grows*, and *Cont.* Based on the order presented in the regression results below, the definition of each control variable is as follows.

The per capital GDP of the host country(*LnGdpp*): the natural logarithm of the host country’s per capital GDP.

Human resource endowment of host country (*LnLAB*): the natural logarithm of the total workforce population of the host country.

Profit margin on corporate net assets (*ROA*): the ratio of after-tax net profit to total assets.

Whether there are foreign executives (*Foreign*): the presence of foreign executive assigned a value of 1, otherwise assigned a value of 0.

Asset-liability ratio of enterprises (*Lev*): the ratio of total assets to total liabilities.

The growth of the enterprise (*Grows*): business revenue growth rate.

Whether it’s a state-owned enterprise (*Cont*): the state-owned enterprise assigned a value of 1, otherwise assigned a value of 0.

See Table 2 for the definition of variables and measurement indexes used in this paper.

**Table 2:** Definition of variables and measurement indexes.

Variable types	Variable names	Measurement
Dependent variable	Enterprises’ green preference of outbound investment (*OIGP*)	By calculating the degree of greenness of each country and weighted by the number of subsidiaries.
Independent variable	Quality of environmental information disclosure(*QEID*)	It consists of 25 indicators from five aspects: environmental management disclosure, environmental information disclosure carrier, environmental certification disclosure, environmental liability disclosure, environmental performance and governance disclosure.
Mediating variables	Green technology innovation(*GTI*)	The number of green inventions authorized by enterprises is measured by adding 1 to the logarithmic value
	Media attention(*MA*)	The total number of news stories that appear on the Internet and in newspapers and magazines each year is divided by 1,000, and the logarithm is taken by adding 1
Control variables	The per capital GDP of the host country(*LnGdpp*)	The natural logarithm of the host country’s per capital GDP
	Human resource endowment of host country (*LnLAB*)	The natural logarithm of the total workforce population of the host country
	Profit margin on corporate net assets (*ROA*)	The ratio of after-tax net profit to total assets
	Whether there are foreign executives (*Foreign*)	The presence of foreign executive assigned a value of 1, otherwise assigned a value of 0
	Asset–liability ratio of enterprises (*Lev*)	The ratio of total assets to total liabilities
	The growth of the enterprise (*Grows*)	Business revenue growth rate
	Whether it’s a state-owned enterprise (*Cont*)	The state-owned enterprise assigned a value of 1, otherwise assigned a value of 0

### Model construction

This paper uses the fixed effect model to examine the impact of QEID on firms’ OIGP and its mechanism. Model 1 is used to test the impact of QEID on enterprises’ green preference in foreign investment. According to the mediation effect analysis method, Models 2 to Model 5 are further constructed to test the mediating effect of GTI and MA.


$$\mathrm{Model}\;1:\;OIGP_{i,t}=\alpha_{0}+\alpha_{1}QEID_{i,t}+\alpha_{2}\sum \mathrm{Control}s_{i,t}+\varepsilon_{i,t}$$

$$\mathrm{Model}\;2:\;GTI_{i,t}=\beta_{0}+\beta_{1}QEID_{i,t}+\beta_{2}\sum \mathrm{Control}s_{i,t}+\varepsilon_{i,t}$$

$$\begin{array}{c} \mathrm{Model}\;3:\;OIGP_{i,t}=\gamma_{0}+\gamma_{1}QEID_{i,t}+\gamma_{2}GTI_{i,t}\\ \;+\gamma_{3}\sum \mathrm{Control}s_{i,t}+\varepsilon_{i,t} \end{array}$$

$$\mathrm{Model}\;4:\;MA_{i,t}=\beta_{3}+\beta_{4}QEID_{i,t}+\beta_{5}\sum \mathrm{Control}s_{i,t}+\varepsilon_{i,t}$$

$$\begin{array}{c} \mathrm{Model}\;5:\;OIGP_{i,t}=\gamma_{4}+\gamma_{5}QEID_{i,t}+\gamma_{6}MA_{i,t}\\ \;+\gamma_{7}\sum \mathrm{Control}s_{i,t}+\varepsilon_{i,t} \end{array}$$

Among them, *OIGP_i,t_* is the enterprise’ green preference of multinational investment, *QEID* is the quality of environmental information disclosure, $\alpha_{i},\;\beta_{i},\;\gamma_{i}$ is the regression coefficient, $\varepsilon_{i,t}$ is the residual. The mediation effect testing process mainly includes the following three steps:

1. The regression of Model 1 is conducted to determine whether environmental information disclosure (*QEID*) has a significant impact on enterprises’ OIGP, that is, whether the coefficient of *QEID* is significant. If it is significant, the mediation effect will be considered; if it is not, the masking effect will be considered.

2. After regression of Models 2 and 3, if the coefficients of *QEID* and *GTI* are significant, it indicates the existence of indirect effects and proceed to the next step. If at least one of them is not significant, the Bootstrap method will be used for the test. If significant, the next step will be carried out; if not significant, it means that there is no mediation effect.

3. Judging whether $\gamma_{1}$ in Model 3 is significant. If so, continue to judge the sign difference between $\beta_{1}\gamma_{2}$ and $\gamma_{1}$ . The same sign indicates the existence of partial mediation effect, while the different sign indicates the existence of masking effect. If it is not significant, it indicates that *GT*I has a complete mediating effect.

The above *methods* can be used to test Models 1, 4 and 5 to check the mediating effect of media attention (*MA*).

## Empirical results

### Descriptive statistics and correlation analysis

In this study, stata16 was used to make descriptive statistics and correlation analysis of the variables involved, and the analysis results are shown in Table 3. Descriptive statistical results show the mean and standard deviation of each variable. The results show that there is a positive correlation between the quality of environmental disclosure and the enterprises’ OIGP. This verifies the rationality and feasibility of hypothesis H1 in this paper to some extent, but the specific results should be further analyzed in the regression test.

**Table 3:** The results of descriptive statistics and correlation analysis.

Variables									
*OIGP*	1.000								
*QEID*	0.031^**^	1.000							
*LnGDPP*	0.120^***^	0.113^***^	1.000						
*LnLAB*	0.106^***^	0.114^***^	0.999^***^	1.000					
*Foreign*	0.011	0.032^**^	0.105^***^	0.106^***^	1.000				
*Cont*	−0.017	0.217^***^	−0.013	−0.014	−0.030^**^	1.000			
*Grows*	0.003	−0.120^***^	0.045^***^	0.044^***^	0.014	−0.021	1.000		
*Lev*	−0.033^**^	0.200^***^	0.170^***^	0.172^***^	−0.011	0.306^***^	0.066^***^	1.000	
*Roa*	−0.032^**^	−0.003	−0.034^**^	−0.032^**^	0.016	−0.085^***^	0.004	−0.360^***^	1.000
Mean	0.449	1.968	80.646	49.896	0.703	0.295	0.268	0.441	0.040
Standard deviation	0.442	0.969	90.117	55.459	0.457	0.456	0.609	0.196	0.057

* *p* < 0.1;

** *p* < 0.05;

*** *p* < 0.01.

### Baseline regression analysis

For scientific consideration of model selection, this paper uses fixed effect model, random effect model and mixed sample model to test Model 1, so as to judge the suitability of various regression methods for model estimation through statistical test. The regression results are shown in Table 4.

**Table 4:** Results of baseline regression.

Variables	Fixed effect model	Random effect model	Mixed effect model
*QEID*	0.047^***^	0.034^***^	0.020^***^
	(5.679)	(5.195)	(3.441)
*LnGDPP*	0.014^***^	0.031^***^	0.043^***^
	(6.306)	(17.997)	(29.181)
*LnLAB*	−0.022^***^	−0.050^***^	−0.068^***^
	(−6.116)	(−17.672)	(−28.776)
*Foreign*	0.003	0.006	0.002
	(0.196)	(0.483)	(0.126)
*Cont*	0.143^***^	−0.016	−0.018
	(3.327)	(−0.834)	(−1.414)
*Grows*	−0.032^***^	−0.020^**^	−0.003
	(−3.198)	(−2.291)	(−0.291)
*Lev*	0.213^***^	−0.042	−0.127^***^
	(3.456)	(−1.050)	(−3.949)
*Roa*	−0.161	−0.285^***^	−0.305^***^
	(−1.372)	(−2.780)	(−2.949)
*Constant*	0.203^***^	0.396^***^	0.454^***^
	(5.628)	(16.078)	(22.747)
*N*	5,602	5,602	5,602
*R* ^2^	0.037	0.0232	0.147
*F*	20.498		120.796
LM		2280.88	
Hausman	211.04		

* *p* < 0.1;

** *p* < 0.05;

*** *p* < 0.01.

Table 4 shows the regression results of fixed effect, random effect and mixed effect models. The regression results show that the improvement of *QEID* has a positive effect on the enterprises’ *OIGP*. The coefficients of explanatory variables in the three models are 0.047, 0.034 and 0.020 respectively, and the three coefficients are significant at the level of 1%, which proves the validity of hypothesis 1. In order to identify the suitability of these three models, two pairs of tests are carried out. First, the LM test result between the mixed sample regression model and the random effect model shows that the Chi-square value is 2280.88, which rejects the null hypothesis at the significance level of 1%. It shows that the random effect model is superior to the mixed sample model. Then, the Hausman test between the random effects model and the fixed effects model shows that the chi-square value of the test is 89.50, and the null hypothesis is rejected at the significance level of 1%. This shows that the fixed effect model is superior to the random effect model. Therefore, the coefficient 0.0451 of *QEID* in the fixed effect model is the most appropriate to describe the relationship between *QEID* and the enterprises’ *OIGP*.

### Mediating effect analysis

#### From the perspective of green technology innovation

Table 5 further shows the empirical results of Model 2 and Model 3, analyzing the mediating effect of the green technology innovation. The results in the table show that the regression coefficient of *GTI* in model (2) is 0.022 and significant at the 5% level, indicating that the improvement of *QEID* will promote enterprises’ *GTI*. However, the coefficient of *GTI* in Model 3 is not significant, so bootstrap method should be used to test it according to the mediation effect testing process. It is found that the Bootstrap (95%) confidence interval does not contain 0, indicating that there is a significant indirect effect between *QEID*, *GTI* and enterprises’ *OIGP*. Then, coefficient $\gamma_{1}$ is tested. The results in Table 5 show that the regression coefficient of *QEID* in Model 3 is 0.045 and it is significant at 1% level. Therefore, the sign of $\beta_{1}\gamma_{2}$ is opposite to $\gamma_{1}$ . According to the mediation effect testing process, it is proved that the mediating effect of *GTI* is a masking effect.

**Table 5:** Results of mediating regression (*GTI)*.

Variables	Model 2: *GTI*		Model 3: *OIGP*	
	Coefficient	*T*-value	Coefficient	*T*-value
*QEID*	0.022^**^	2.35	0.047^***^	5.68
*GTI*			−0.002	−0.18
*_cons*	0.194^***^	4.72	0.203^***^	5.63
Control variables	Control		Control	
*N*	5,602		5,602	
*R^2^*	0.0120		0.0365	
*F*	6.59		18.22	

* *p* < 0.1;

** *p* < 0.05;

*** *p* < 0.01.

The regression results show that *GTI* plays a mediating role that is inconsistent with hypothesis 2 of this paper, where an increase in the level of *GTI* decreases firms’ *OIGP,* and thus masking part of the *QEID’s* contribution to firms’ *OIGP*. This inhibitory effect may be due to the existence of “crowding-out effect,” that is, the increase in QEID would lead to more intensive investment of funds and talents in green technology innovation projects. Based on the “crowding-out effect” and the “pollution refuge hypothesis” rational manufacturers will prefer to invest in areas with low environmental costs, less stringent environmental regulations in order to balance total costs, thus avoiding the pressure of environmental regulations and achieving profit maximization (Bommer, 1999). Besides, most of the literature assumes that the R&D investment of enterprises is a high-quality activity, which can naturally greatly improve the production efficiency of enterprises. However, Li and Zheng (2016) holds that we can classify firms’ innovation behavior into substantive and strategic innovation based on whether the purpose of innovation is real, and their empirical results show that substantive innovation oriented to technological progress is the source of firm value, while strategic behavior that unilaterally pursues the increase in the number of patents is not significantly related to firm value. The green innovation research and development stimulated by *QEID* may include some “fake” innovations such as strategic innovations. Therefore, although the level of green innovation does increase, it does not contribute to the green preference of foreign investment. At the same time, due to the increase in R&D costs, green innovation may inhibit the green preference of foreign investment. Although *GTI* has a restraining effect on enterprises’ OIGP, this restraining effect cannot completely mask the positive effect of *QEID* on corporate *OIGP*, so in general *QEID* is still reflected as a promoting effect on enterprises’ *OIGP*.

#### From the perspective of media attention

Table 6 shows the empirical test results of Model 4 and Model 5. The results in the table show that the regression coefficient of media attention (*MA*) in Model 4 is −0.013 and significantly negative at the 1% level, indicating that an increase in the quality of environmental information disclosure inhibits media attention to the company. In Model 5, the regression coefficient of *MA* is −1.44 and is significant at the 1% level, while the regression coefficient of *QEID* is 0.043 and significant at the 1% level. According to the mediation effect test process, when $\beta_{4}$ , $\gamma_{5}$ , and $\gamma_{6}$ are significant, the same sign of $\beta_{4}\gamma_{6}$ and $\gamma_{5}$ indicates that there is a mediating effect of media attention between *QEID* and enterprises’ *OIGP*. The improvement of environmental information disclosure will promote the *OIGP* of enterprises by suppressing media attention, thus hypothesis 3 holds.

**Table 6:** Results of mediating regression (MA).

Variables	Model 4: *MA*		Model 5: *OIGP*	
	Coefficient	*T*-value	Coefficient	*T*-value
*QEID*	−0.013^***^	−3.43	0.045^***^	5.44
*MA*			−0.154^***^	−4.64
*_cons*	0.360^***^	21.82	0.258^***^	6.82
Control variables	Control		Control	
*N*	5,602		5,602	
*R^2^*	0.0179		0.0413	
*F*	9.89		20.70	

* *p* < 0.1;

** *p* < 0.05;

*** *p* < 0.01.

**Figure 1:**
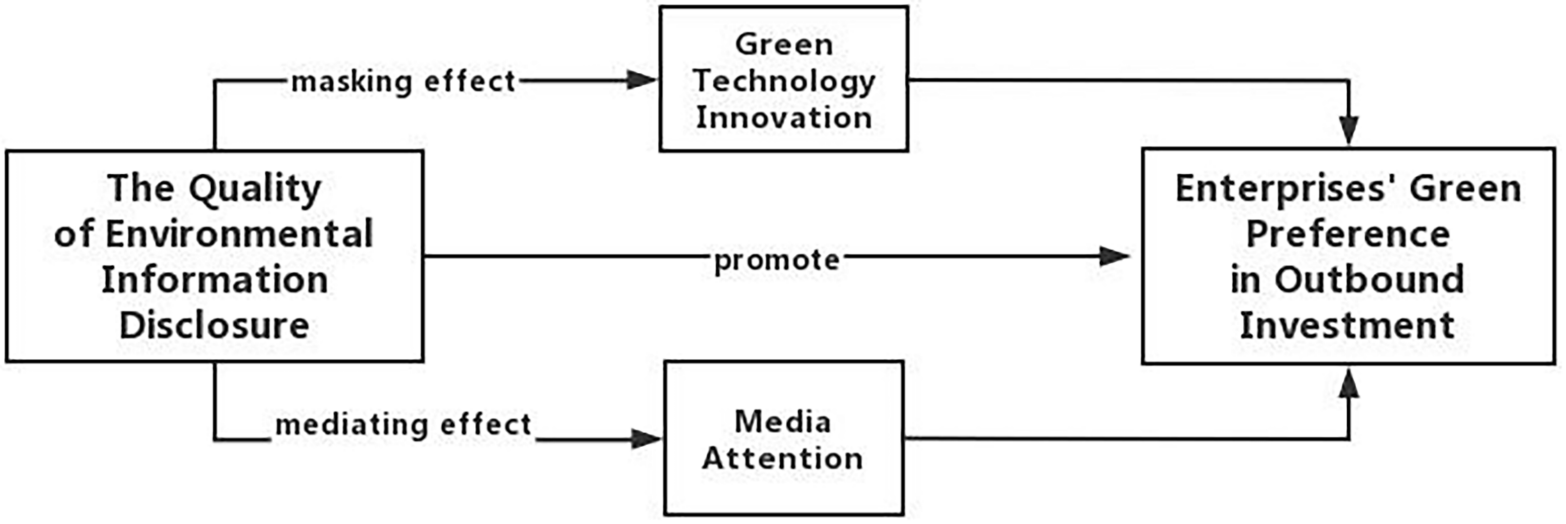
Theoretical analysis model diagram.

The above regression results illustrate how *QEID* affects enterprise’s *OIGP* and illustrate the specific mediating influence mechanism. This allows us to obtain a conceptual map of the model, as shown in Figure 1.

## Heterogeneity tests

### Analysis from the perspective of different property rights

In order to investigate the difference of *QEID* on *OIGP* under different property rights, this paper divides the sample into state-owned and non-state-owned enterprises for regression. The results are shown in Table 7.

**Table 7:** Regression results of different property rights.

Variables	Group A: State owned enterprise					Group B: Non-state owned enterprise				
	Model 1	Model 2	Model 3	Model 4	Model 5	Model 1	Model 2	Model 3	Model 4	Model 5
*QEID*	0.052^***^	0.032	0.053^***^	−0.008	0.052^***^	0.042^***^	0.024^**^	0.042^***^	−0.015^***^	0.039^***^
	(3.58)	(1.54)	(3.58)	(−0.96)	(3.52)	(4.18)	(2.39)	(4.18)	(−3.57)	(3.92)
*GTI*			−0.001					−0.001		
			(−0.05)					(−0.08)		
*MA*					−0.117^**^					−0.175^***^
					(−2.35)					(−3.90)
Cons	0.315^***^	0.284^**^	0.315^***^	0.480^***^	−0.371^***^	0.256^***^	0.105^***^	0.256^***^	0.282^***^	0.305^***^
	(3.94)	(2.52)	(3.93)	(10.82)	(4.46)	(6.87)	(2.83)	(6.86)	(18.55)	(7.77)
Control variables	Control	Control	Control	Control	Control	Control	Control	Control	Control	Control
*N*	1,652	1,652	1,652	1,652	1,652	3,950	3,950	3,950	3,950	3,950
*R^2^*	0.0305	0.0432	0.0305	0.0128	0.0345	0.0393	0.0071	0.0393	0.0242	0.0442
*F*	5.89	8.46	5.15	2.44	5.86	17.30	3.02	15.14	10.51	17.11

* *p* < 0.1;

** *p* < 0.05;

*** *p* < 0.01.

In both groups of state-owned (SOE) and non-state-owned (non-SOE) enterprises, the coefficient of *QEID* in Model 1 is significantly positive at the 1% level, which remains consistent with Hypothesis 1. This indicates that an increase in *QEID* promotes firms’ *OIGP*, whether in state-owned or non-state-owned enterprises. The next step is to examine the mediating effects played by *GTI* and MA in SOEs and non-SOEs. First, the mediating variable *GTI* is tested, and the coefficients of *QEID* in Model 2 and GTI in Model 3 in group A are not significant, while the coefficient of *GTI* in Model 3 in group B was also not significant, so this mediation mechanism will be tested using Bootstrap according to the mediation test procedure. The test results showed that the (95%) confidence interval of Bootstrap test for *GTI* in both groups A and B did not contain zero, and based on the sign of the coefficients, we can judge that the mediating effect of *GTI* in both groups A and B still showed a masking effect. However, heterogeneity in the results between state-owned and non-state-owned firms emerges when the mediating variable of *MA* is examined. The mediating effect played by media attention in the non-state group as seen in Table 6 remains consistent with the previous section. However, in the sample of SOEs, the coefficient of *QEID* of model 4 is not significant. It is further tested using bootstrap test and the results show that the mediating effect of *MA* in SOEs is not significant.

In summary, the difference in the nature of property rights is mainly reflected in the mediating variable of *MA*. This may be due to the special property rights of SOEs, which make their market competition mechanism weaker leading to the failure of market pressure mechanism, thus weakening the monitoring role of media attention (Zeng et al., 2016). At the same time, reputation is very important for political promotion of management in SOEs, and public opinion pressure has a serious impact on their promotion. They will maintain a good reputation by strengthening internal control construction (Pan et al., 2019). Therefore, state-owned enterprises rarely have negative news, which leads to a relatively weak influence of public opinion on them. In the study on the heterogeneity of the nature of property rights, Wang et al. (2021) also find that non-SOEs are more susceptible to negative media coverage, so it is not difficult to understand that media attention does not exert its mediating effect in SOEs.

### Analysis from the perspective of different life cycle stages

Based on the practice of Liu et al. (2020), we defines life cycle stages of enterprises with different combinations of operating cash flow, investment cash flow, and financing cash flow. This method has a strong operability and objectivity. In this paper, listed enterprises are divided into three major stages: growth, maturity and decline, as a way to test the heterogeneous impact of *QEID* on corporate *OIGP* in different life cycle stages.

The regression results of these three subgroups are presented in Table 8, and the results of Bootstrap test are presented in Table 9. Table 8 shows that the regression coefficients of *QEID* in model (1) in all three groups are significantly positive, and the *QEID* of firms in different life cycle stages will promote *OIGP*, and this promotion effect is the most significant in the mature stage and the weakest in the declining stage. This result may be due to the fact that enterprises in the maturity stage are more mature in production and operation and have basically achieved stable profitability. At the same time, enterprises in this period have already established their market reputation and can obtain much more external financial support at a lower cost (Huang et al., 2016). Enterprises would have more sufficient funds for foreign investment at this time, and at the same time, the goal of enterprises in this period has shifted from short-term profit maximization to long-term profit maximization of enterprises, and their social goals are more ambitious (Li and Hu, 2008). Enterprises in this stage will have a stronger awareness of environmental protection and sustainable development, so they will be more inclined to invest in countries with a high degree of green. For enterprises in decline, there are often problems such as poor innovation ability, institutional rigidity and shirking responsibility of the management (Li et al., 2011). At the same time, they also face poor financial conditions in this stage. So, enterprises tend to choose a conservative business strategy in this stage. This may lead to management short-sightedness and a decline in environmental consciousness. Consequently, *QEID* is less significant in promoting *OIGP* in enterprises in decline stage than those in mature and growth stages.

**Table 8:** Regression results of different life cycle stages.

Group A: Growth stage	Model 1	Model 2	Model 3	Model 4	Model 5
*QEID*	0.028^**^	0.022	0.028^**^	−0.010^*^	0.026^**^
	(2.22)	(1.47)	(2.22)	(−1.68)	(2.07)
*GTI*			0.001		
			(0.01)		
*MA*					−0.184^***^
					(−3.83)
*Cons*	0.200^***^	0.127^*^	0.200^***^	0.397^***^	0.273^***^
	(3.65)	(1.64)	(3.43)	(14.37)	(4.72)
Control variables	Control	Control	Control	Control	Control
*N*	2,775	2,775	2,775	2,775	2,775
*R* ^2^	0.0454	0.0171	0.0454	0.0183	0.0535
*F*	10.17	3.72	9.03	3.99	10.74
Group B: Maturity stage	Model 1	Model 2	Model 3	Model 4	Model 5
*QEID*	0.058^***^	0.022	0.058^***^	−0.006	0.058^***^
	(3.31)	(1.02)	(3.30)	(−0.75)	(3.29)
*GTI*			0.009		
			(0.36)		
*MA*					−0.067
					(−1.01)
*Cons*	0.218^**^	0.183^*^	0.216^**^	0.348^***^	0.241^***^
	(2.54)	(1.76)	(2.51)	(8.62)	(2.71)
Control variables	Control	Control	Control	Control	Control
*N*	1,938	1,938	1,938	1,938	1,938
*R* ^2^	0.0321	0.0237	0.0323	0.0281	0.0331
*F*	4.32	3.16	3.85	3.75	3.95
Group C: Decline stage	Model 1	Model 2	Model 3	Model 4	Model 5
*QEID*	0.053^*^	−0.001	0.053^*^	−0.020^*^	0.050^*^
	(1.91)	(−0.05)	(1.91)	(−1.87)	(1.79)
*GTI*			0.033		
			(0.55)		
*MA*					−0.157
					(−1.09)
*Cons*	0.268^**^	0.248^**^	0.260^**^	0.299^***^	0.315^***^
	(2.43)	(2.43)	(2.34)	(7.16)	(2.66)
Control variables	Control	Control	Control	Control	Control
*N*	889	889	889	889	889
*R* ^2^	0.0759	0.0080	0.0768	0.0610	0.0792
*F*	3.42	0.34	3.07	2.70	3.17

* *p* < 0.1;

** *p* < 0.05;

*** *p* < 0.01.

**Table 9:** Bootstrap test results.

Variables	Group A: Growth stage		Group B: Maturity stage		Group C: Decline stage	
	Lower limits	Upper limits	Lower limits	Upper limits	Lower limits	Upper limits
*GTI*	−0.0078	−0.0025	−0.0060	−0.0006	−0.0039	0.0017
*MA*			−0.0018	0.0034	−0.0040	0.0012

The mediating effect of *GTI* and MA in these three periods of the firm is further examined by combining Bootstrap test. The mediating effect of *MA* in group A remains consistent with the previous paper, but the mediating effect of *MA* in two groups, B and C, is not significant. This may be because the growth period is a critical period for enterprises to build their corporate image, so *MA* has a much greater impact on enterprises in the growth period compared to the other two periods. The test results of *GTI* show that the mediating effect of *GTI* in mature firms is consistent with the previous empirical evidence, but *GTI* in mature firms shows a partial mediating effect, which means that the *QEID* of mature firms will improve their *OIGP* by promoting *GTI.* Meanwhile, the mediating effect of *GTI* is not significant in enterprises in decline stage. This may be due to tighter financing constraints, higher capital expenditures, lack of R&D experience, and low innovation success when the firm is in the growth stage, but these conditions change significantly when the firm is in the maturity stage (Liu et al., 2020). Enterprises in mature stage are experienced in R&D and tend to invest in projects with high future returns or in invention patents. At the same time, years of operation make enterprises in mature stage have a certain amount of capital accumulation. Therefore, the “crowding out effect” is more obvious in growth enterprises than in mature enterprises. In addition, the percentage of “spurious” innovations, such as strategic technological innovations, is likely to be lower in mature enterprises than in growth enterprises. Therefore, *GTI* can play a positive partial mediating effect in mature enterprises as described in Hypothesis 2. In contrast, enterprises in decline stage face deteriorating financial conditions, difficulties in raising funds, and a significant decline in innovation awareness, which leads them to be reluctant to invest much in R&D projects. Therefore the mediating effect of *GTI* in enterprises in decline stage is not significant.

## Robustness tests

### Propensity score matching

In this paper, we use the PSM method to control the endogeneity problem, and simultaneously choose three ways to test: the nearest neighbor matching, radius matching and kernel matching. First, the QEID of each year in the sample is assigned a value of 1 if it is higher than the average, and 0 if it is not. The selected matching variables are the control variables in the previous section. The two sets of samples are then mixed and random seeds are generated. The results are shown in Table 10. The average treatment effect before matching is 0.036 and significant at the 1% level, indicating that the quality of corporate green disclosure without considering the control variables increases green preferences of outbound investment by 3.6%. The ATT after using nearest neighbor matching is 0.038 and is significant at the 1% level. This represents a 3.8% increase in OIGP for firms with high QEID compared to firms with low QEID. Further replacing the nearest neighbor matching with radius matching and kernel matching, the results of all three matching methods are significant and the average treatment effect is similar. The robustness of the conclusion in this paper is demonstrated.

**Table 10:** PSM test results.

	Treated	Controls	ATT
Unmatched	0.467	0.431	0.036^***^
Nearest neighbor matching (1:1)	0.463	0.431	0.032^***^
Radius matching (0.001)	0.464	0.425	0.038^***^
Kernel matching	0.464	0.425	0.038^***^

* *p* < 0.1;

** *p* < 0.05;

*** *p* < 0.01.

### Placebo test

**Figure 2:**
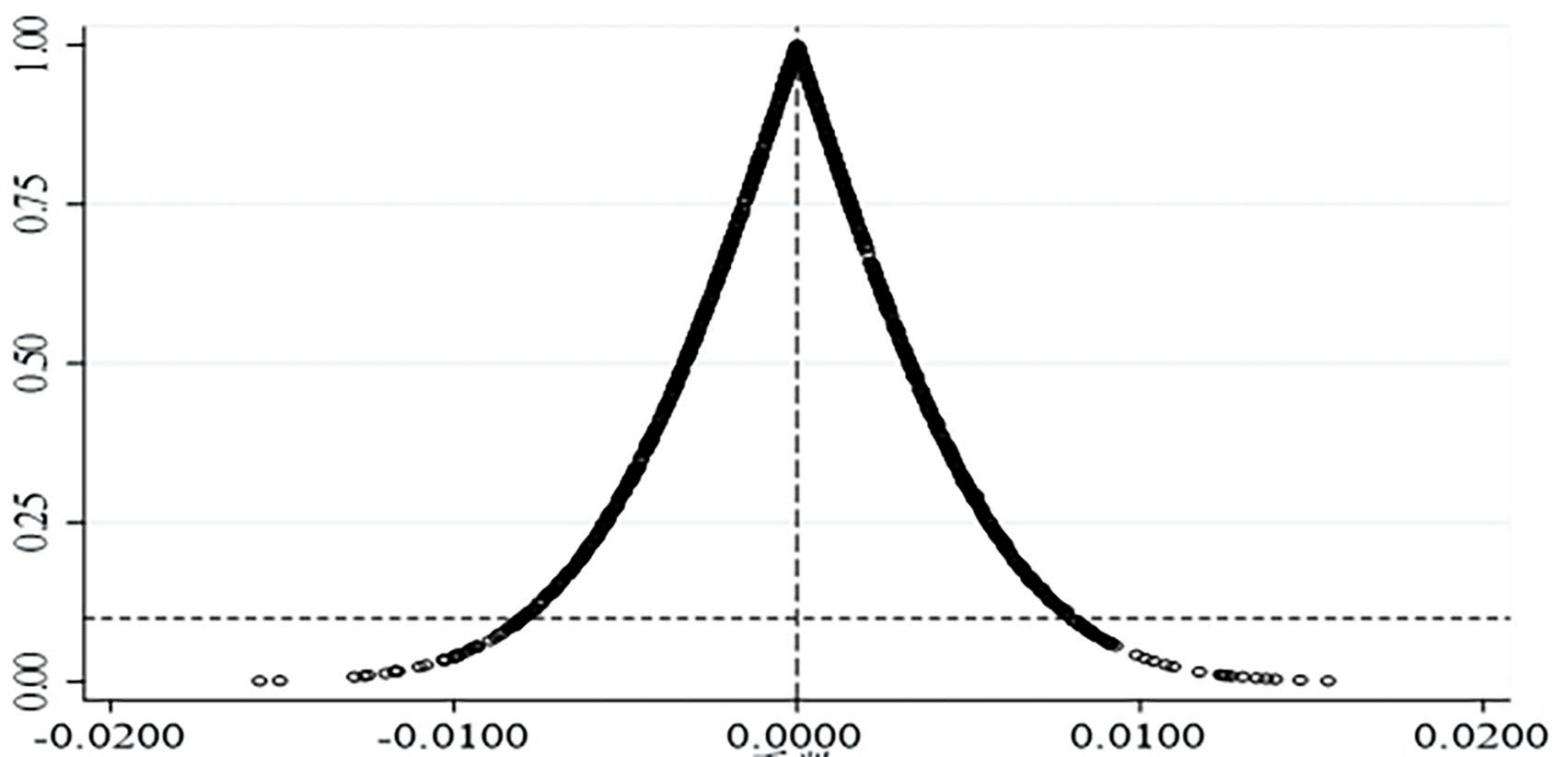
Statistical results of coefficient and *p* value in placebo test. The horizontal axis represents the coefficient and the vertical axis represents the value of *p*.

The positive relationship between QEID and the firm’s OIGP may be confounded by omitted variables, so this paper uses a placebo test to eliminate the interference of omitted variables on the conclusion. The QEIDs are first randomly swapped in the listed companies, then matched with other variables, and finally substituted into Model 1 for regression. The results are shown in Panel A in Table 11. If the positive correlation between QEID and firms’ OIGP is due to the omitted variable, the result will still be significantly positive after re-matching. However, the results in the table show that the coefficient of QEID is negative and no longer significant after random transformation, indicating that the finding that QEID promotes firm’s OIGP is not affected by the omitted variable. To further enhance the robustness of the placebo test results, in this paper, the coefficients and *p*-values of the regressions are tallied after the above steps were repeated 1,000 times. The statistical results are shown in Figure 2. It can be seen from the figure that the percentage of QEID’s coefficients that are significantly positive after 1,000 repeated regressions is very small, implying that the virtual treatment effect constructed in this paper does not exist, thus proving the robustness of the conclusions of this paper.

**Table 11:** Robustness test results.

	Panel A: *OIGP*	Panel B: *OIGP*
*QEID*	−0.003	0.021^***^
	(−0.60)	(2.63)
*Cons*	0.245^***^	0.265^***^
	(6.89)	(7.83)
Control variables	Control	Control
*N*	5,602	5,602
*R^2^*	0.0368	0.0309
*F*	18.35	17.23

* *p* < 0.1;

** *p* < 0.05;

*** *p* < 0.01.

### Alternative explanatory variable

This paper draws on the study of Xu et al. (2021) to measure the QEID of listed companies in three dimensions: environmental protection philosophy, environmental protection objectives, and environmental management system. Three dummy variables are set according to whether listed companies have disclosed information on these three aspects in the current year, and QEID is set as the sum of these three dummy variables (taking values between 0 and 3) to replace the explanatory variables in the previous paper. The regression results are presented in Panel B of Table 11. The regression results of model (1) are still significantly positive at the 1% level after replacing the explanatory variables, indicating that QEID still promotes enterprises’ OIGP.

### Further control other control variables

Some other host country macro-environmental characteristics and firm-level factors may also affect firms’ outbound investment decisions, and for this reason this paper further controls for other potentially missing variables, including host country-level technological resource endowment *(LnPatent*) and natural resource endowment (*Raw*), as well as firm-level dual job (*Dual*), labor-to-capital ratio (*Alab*), and management shareholding ratio (*Mshare)*. The results are shown in Table 12. The regression results of Model 1, Model 2, Model 4, and Model 5 are consistent with the previous paper except for the insignificant coefficient of *GTI* in Model 3. The mediation effect of *GTI* is tested using Bootstrap according to the mediation test procedure. The test results show that its 95% confidence interval does not contain 0. Therefore, the mediating effect of *GT*I exists and still plays a masking effect.

**Table 12:** Results after controlling other variables.

	Model 1	Model 2	Model 3	Model 4	Model 5
*QEID*	0.042^***^	0.018^*^	0.043^***^	−0.012^***^	0.041^***^
	(5.16)	(1.91)	(5.17)	(−3.13)	(4.96)
*GTI*			−0.005		
			(−0.40)		
*MA*					−0.143^***^
					(−4.34)
Cons	0.193^***^	0.226^***^	0.194^***^	0.358^***^	0.245^***^
	(4.77)	(4.88)	(4.79)	(19.27)	(5.81)
Control variables	Control	Control	Control	Control	Control
*N*	5,602	5,602	5,602	5,602	5,602
*R* ^2^	0.0488	0.0162	0.0488	0.0273	0.0529
*F*	17.05	5.47	15.84	9.33	17.24

* *p* < 0.1;

** *p* < 0.05;

*** *p* < 0.01.

### Two-stage least square

In view of the fact that firms may improve the quality of their environmental information disclosure in the process of outbound investment in order to meet international requirements for corporate social responsibility, it leads to the possibility that the main model regression results may be affected by endogenous problems. In this paper, the explanatory variable lagged one period (*QEID_t–1_*) is used as an instrumental variable to perform robustness tests using two-stage least squares (2SLS) by referring to the research method of Xi and Zhang (2022). The results of the 2SLS are shown in Table 13, where column (1) shows the results of the first stage regression of the instrumental variable. The coefficient of *QEID_t-1_* in the first stage is significantly positive, and the regression result is in line with expectation. Meanwhile, the *F*-value of the first stage is greater than 10, indicating that the instrumental variable is selected appropriately. Column (2) shows the regression results for the second stage of the instrumental variable, which remain significantly positive, indicating that the main finding of this paper remain robust after considering the potential endogenous problems of the main regression model.

**Table 13:** Results of 2SLS.

Variables	First stage: *QEID*		Second stage: *OIGP*	
	Coefficient	*T*-value	Coefficient	*T*-value
*QEID_t–1_*	0.270^***^	11.52		
*QEID*			0.236^***^	5.47
Control variables	Control		Control	
*N*	3,821		3,821	
*F* (first stage)	132.73			
C.D Wald *F*				
K.P. Wald *F*				

## Conclusion and discussion

### Conclusion

This paper selects the outbound investment events of Chinese listed companies from 2008 to 2019 as a research sample to study the impact of the QEID on enterprises’ OIGP from the micro perspective of firms. The empirical results show that, in general, the increase of QEID promotes enterprises’ OIGP. In terms of the influence mechanism, GTI shows a masking effect in QEID promoting enterprises’ OIGP, while media attention plays a partial mediating effect in QEID promoting enterprises’ OIGP.

At the same time, this paper finds in further research that this influence mechanism varies among enterprises with different property rights and different life cycle stages. (1) The main effect remains consistent across enterprises with different ownership properties, but media attention plays a mediating effect only in non-state-owned enterprises, while the mediating effect is not significant in state-owned enterprises. (2) Among the firms in different life cycle stages, the QEID of mature stage firms has the most significant contribution to their OIGP, and the weakest in the decline stage. The mediating effect of GTI in enterprises at different life cycle stages also differs. It shows a masking effect in growth stage enterprises, a partial mediating effect in mature stage enterprises, and a non-significant mediating effect in declining stage enterprises.

This paper takes environmental information disclosure as the starting point and constructs a theoretical framework of QEID affecting enterprises’ OIGP based on existing frontier theories and advanced experiences, and confirms that QEID improves enterprises’ OIGP, which provides empirical evidence and practical reference for enterprises to optimize green preference of outbound investment and implement green sustainable development strategy. The establishment of the explanatory mechanism of the relationship between QEID and enterprises’ OIGP helps to deeply understand the internal logic of enterprises’ social responsibility influencing their outbound investment behavior, and enriches the research on green location choice of enterprises’ outbound investment. The above findings help to understand the role of QEID in corporate governance and provide important insights for listed companies to strengthen their responsibility for environmental protection, further integrate economic development into ecological environmental protection, and optimize their foreign investment behavior so as to ultimately achieve healthy and sustainable economic development.

### Policy suggestions

This study is intended to make the following policy suggestions for the purpose of the implementation of green development and optimization of the foreign investment behavior of enterprises.

For enterprises: (1) Enterprises should actively implement the concept of “energy saving, emission reduction and green development” promote the environmental information disclosure, and try to improve the quality of environmental information disclosure. (2) Enterprises should actively carry out substantive technological innovation, especially increase investment in green innovation projects, and make full use of policy support to improve their green innovation efficiency. Enterprises especially those in the growth stage should improve their governance structure and eradicate the short-sighted behavior in decision-making, so as to ensure strong capital guarantee, talent guarantee and mechanism guarantee for green technology innovation, thus providing important technological support for their green outbound investment. (3) Media attention is an effective external governance mechanism, which can help enterprises improve internal governance and management efficiency. Therefore, in the face of high-intensity media attention and public opinion pressure, enterprises especially non-state-owned enterprises should take a positive attitude and strive to focus on improving the internal governance quality to ultimately provide effective support for enterprises’ green outbound investment and improve the long-term value of enterprises. (4) Finally, when enterprises make outbound investments, they should conduct in-depth observation and extensive understanding of the host country’s risks, resource endowment, environmental regulations and entry costs in advance, and make the most objective decisions by making a comprehensive assessment of the host country. Enterprises should make differentiated outbound investment decisions based on different property rights and different life cycle stages of enterprises, and choose appropriate host countries as their investment destination.

For government agencies: (1) Relevant government agencies should try to release relevant reliable,complete and timely information concerning environment and risks of host countries. (2) Relevant government agencies should adopt appropriate policies to encourage enterprises especially growth enterprises to carry out green technology innovation, effectively distinguish between substantive innovation and strategic innovation, and increase support for substantive innovation, thereby improving green innovation efficiency, reducing the masking effect of green technology innovation, and strengthening its supporting role for green outbound investment of enterprises. (3) Relevant government agencies should try their best to improve the supervision mechanism of the media in relevant economic fields, guide the media and public opinion to play a supervisory role in the operation of various enterprises, ensure the authenticity, timeliness and reliability of media reports, and effectively play its role in external governance of enterprises. (4) Finally, due to the uneven quality of environmental information disclosure of listed companies in China, relevant government agencies should consider establishing a more stringent mandatory green information disclosure system, gradually promoting the transformation of green information disclosure from qualitative to quantitative, and establishing an effective environmental information disclosure quality evaluation system to more accurately assess the green performance of enterprises and promote green outbound investment of enterprises.

### Limitations and further research

There are still some limitations in this paper. First of all, the study in this paper is based on a sample of Chinese enterprises, and the conclusions inducted may not be applicable to other countries. In the future, we can expand the research sample from Chinese enterprises to enterprises in more countries, and appropriately extend the sample time span, which will help to obtain more general conclusions. It is also interesting to compare China with other countries and explore whether the impact of QEID on the OIGP of enterprises in other countries is the same as that in China. If different, what are the reasons for such differences? These are worthy of future in-depth studies. Secondly, in this paper, the main consideration is the influence of QEID on the OIGP of enterprises. However, there are many factors that influence enterprises’ outbound investment decisions, so the theoretical system of green location selection for enterprises’ outbound investment can be enriched by considering other characteristics of enterprises in the future. Finally, due to the difficulty of data acquisition, this paper measures the green degree of the host country based on the EPI index, which may not be comprehensive and objective enough. In the future, we can construct more diversified and comprehensive indicators to measure the green degree of the host country.

## Data availability statement

Publicly available datasets were analyzed in this study. This data can be found at: the Chinese CSMAR database (https://cn.gtadata.com), the Chinese CNRDS database (https://www.cnrds.com), and the World Bank database (https://data.worldbank.org.cn/).

## Author contributions

All authors listed have made a substantial, direct, and intellectual contribution to the work and approved it for publication.

## Funding

This study was supported by Chongqing Federation of Social Sciences, “The research on the impact of accounting standards difference on the two-way interactive investment between Chongqing and the ‘the Belt and the Road’” under Grant (number 2018YBJJ032) and Sichuan International Studies University, “The theoretical and empirical research on the influence of environmental information disclosure on firms’ green preferences for foreign investment” under Grant (number SISU2022YZ009).

## Conflict of interest

The authors declare that the research was conducted in the absence of any commercial or financial relationships that could be construed as a potential conflict of interest.

## Publisher’s note

All claims expressed in this article are solely those of the authors and do not necessarily represent those of their affiliated organizations, or those of the publisher, the editors and the reviewers. Any product that may be evaluated in this article, or claim that may be made by its manufacturer, is not guaranteed or endorsed by the publisher.
